# Pipeline Programs Can Support Reforms in Medical Education: A Cohort Study of Alabama's Rural Health Leaders Pipeline to Engage Community Leaders

**DOI:** 10.1111/jrh.12531

**Published:** 2020-11-06

**Authors:** John R. Wheat, James D. Leeper

**Affiliations:** ^1^ College of Community Health Sciences The University of Alabama Tuscaloosa Alabama

**Keywords:** county leaders, medical education reform, pipeline, rural physicians

## Abstract

**Purpose:**

To demonstrate for county leaders the utility of rural pipelines to gain physicians and produce health professionals.

**Methods:**

This cohort study, 1993‐2018, aggregated 1,051 students in the Rural Health Leaders Pipeline to their home counties (N = 67) to study the relationship between county participation in pipeline programs and outcomes of family physicians gained and health professionals produced. Additional county demographics were included. We conducted descriptive, bivariate, and multivariable linear regression analyses controlling for poverty, race, and rurality.

**Findings:**

All 67 Alabama counties participated with means of 9.6 Rural Health Scholars, 2.7 Rural Minority Health Scholars, 3.4 Rural Medical Scholars, 67% rural population, 29.7% Black population, and 21.5% under poverty. Best regression model for gaining family physicians included Rural Medical Scholars involved (*b* = 0.24, *P* < .001) with *R* ^2^ 0.30, indicating a county gained 1 family physician for 4 students. Best model for health professionals included Rural Health Scholars involved (*b* = 0.20, *P* < .001) with *R* ^2^ 0.31, indicating production of 1 health professional for 5 students. Best model for any professional included Rural Health Scholars involved (*b* = 0.23, *P* < .001) with *R* ^2^ 0.35, indicating 1 professional produced for 4 students.

**Conclusions:**

Rural pipeline programs can be useful tools in medical education reform to benefit counties with the gain of family physicians and production of health professionals. Local public officials could use these findings, eg, 1 family physician gained for every 4 students a county involved in the pipeline, to advocate that health professional education employ such pipelines.

The World Health Organization,^1^, ^2^ philanthropies,^3^, ^4^ and medical education leaders^5^, ^6^ call for reforms in medical education. They envision training and dispersing more primary care physicians across countries, regions, and communities to achieve equitable care and healthier populations. Their central tenets are expanding medical classes and adapting admissions, learning contexts, and curricula to match population needs. Reformers address rural and underserved populations, diversified student bodies, and interprofessional training and advocate involving public leadership to help avoid expansion without including other necessary reforms. Medicals schools have increased in size and new schools have been added—21 as of 2018.^7^ However, the number of physicians choosing rural practice still declines, while choice of urban practice increases. Decrease in rural‐origin student numbers is a factor.^8^

Causes for rural health care shortages go beyond student backgrounds. A comprehensive review identified 6 categories of reasons—physical/infrastructural, professional, educational, sociocultural, economic, and political—and attributed failure to advance rural health care, in part, to metrocentric political environments affecting funding, regulatory policies, and cultural sensitivity.^9^ The authors of this paper have observed reluctance among civic and political leaders to engage in deliberations about medical education beyond superficially stating their perception of needs. Local public leadership in the United States, as elsewhere,^10^ has not engaged in a way to assure that reforms are effective for producing more rural physicians.

“Rural pipeline” is a way to increase rural health professionals, acting through the processes of making career choices, attaching to places, and taking up and remaining in rural practice.^11^ “Pathways to rural practice” has similar application referring to processes in 4 stages: (1) before admission to medical school; (2) during medical education for the medical degree; (3) during postgraduate residency training; and (4) continuing medical education after residency.^12^ Rural medical educators facilitate these processes by implementing programs throughout this educational continuum.^13^ Rural medical programs typically aim to produce rural primary care physicians by involving students from rural backgrounds, educating them in family medicine, and using rural community settings.^14^ Pipeline applications have started at k‐12 and have included preadmissions, admissions, curricular, and training programs among professional schools. Some starting in high school have continuing components in medical education, eg, the University of Louisville, Trover program.^15^ Pipeline programs have reported success with outcomes including attitudes, intentions, admission, performance, specialty choice, and practice location.^16^ Those evaluations took institutional perspectives that may not resonate with local community leaders. It is important that programs demonstrate and articulate the value of medical education reforms that construct pipeline programs in ways that engage these leaders and their influence in political processes. Australia's conceptualization of the “rural generalist pathway” models comprehensive multifaceted efforts required to develop a system to produce rural physicians to sustain health care and contribute to the health and well‐being of Australia's rural populations.^10^, ^17^

This paper's intent is to engage county leaders in assessing rural pipeline programs by taking a community perspective that uses county as the unit of observation. We seek to demonstrate and articulate the usefulness of the University of Alabama (UA) Rural Health Leaders Pipeline (RHLP) to enhance engagement of local public leaders.

## Methods

### Setting

Alabama consists of 67 counties; two‐thirds are both rural and chronically underserved with primary care, as demonstrated in primary care health professional shortage area maps, eg, http://www.alabamapublichealth.gov/ruralhealth/Primary%20Care%20HPSA%20Map%20-%20October%202017.pdf. Among the latter are Alabama's Black Belt counties that are known for their rich dark soil, agricultural heritage, and large African American population.^18^ The authors initiated the UA RHLP to increase the number of rural students becoming rural health professionals.^19^ We proposed that rural high school students nurtured through a pipeline program, and others who joined downstream, would benefit themselves and rural counties by becoming health professionals. The State of Alabama funded 2 preprofessional pipeline component programs (described below) at $1,500 per student per year. Students in the medical school component could compete for full tuition scholarships made available by the state to any medical student committing to rural practice, depending on yearly availability of funds. We collected data through 25 years of work with the RHLP.

RHLP includes 3 programs, 1 each at high school, posthigh school, and graduate/ professional school levels. RHLP recruits rural Alabama students in partnership with local schools, colleges, and health care organizations. Students whose homes and schools are outside the major population centers of Alabama and who self‐identify as rural are eligible for consideration. We select students based on personal statement of interest, academic standing, and recommendations. The Rural Health Scholars Program (RHS) takes students who have completed the 11th grade for a summer term at UA for college credit and orientation to health careers. The Rural Minority Health Scholars Program (RMHS) provides a similar experience for minority students in the summer after 12th grade. The Rural Medical Scholars Program (RMS) is a 5‐year program combining rural community health (either certificate or master's degree) and medical studies with rural emphasis at a clinical branch campus of the University of Alabama School of Medicine. We evaluated component programs’ processes and outcomes^14^, ^19^, ^20^, ^21^, ^22^ finding positive results; however, we had not taken an overview of the pipeline for responsiveness to rural counties.

We started this project with the a priori hypothesis that counties’ participation in RHLP programs correlates with gain of physicians and production of other professionals. Related study questions were:

1. What county characteristics correlate with production of professionals and gain of family physicians from RHLP programs?
2. What association does each RHLP program have with county production of professionals and gain of family physicians after county characteristics have been considered?

This observational cohort study aggregated data to the county level to assess county use of 3 RHLP programs and outcomes of health professionals produced. The UA Institutional Review Board authorized the study as exempt (IRB # EX‐18‐CM‐075‐R1). We used the STROBE cohort checklist when writing our report.^23^

### Study Population

The subject population was counties of Alabama, including urban counties because rural parts of these counties provided some students. Counties were characterized with information from RHLP programs supplemented by public data. We collected data from the start of each program—RHS from 1993 to 2017, RMHS 2000 to 2017, and RMS 1996 to 2017. We deidentified and aggregated students to their counties of origin, providing number of students in each RHLP program as measures of county participation (eg, intervention variables). Public data were census‐derived county demographics and Alabama Rural Health Association county classifications.^24^, ^25^

### Outcomes

We counted for each county the total professionals produced (TOTPROF), health professionals produced (HPROF), and family physicians from the pipeline that entered the county (FP). These outcomes reflected the time delay from students beginning a program until entering into professional practice. With family physicians, these delays were a minimum of 12, 11, and 8 years for RHS, RMHS, and RMS, respectively. For RHS, only students entering 1993‐2005 could contribute to FP; the remainder were still in the pipeline (or took other paths). For RMHS, students entering 2000‐2006 could contribute to FP. For RMS, students entering 1996‐2011 could contribute to this outcome. Table 1 presents exposure and outcome data.

**Table 1:** Distribution Among 67 Counties of Program Outcome and Exposure Occurrences

		Occurrences Among Counties							
Outcomes	Definition		Col. 1	Col. 2	Col. 3	Col. 4	Col. 5	Col. 6	Total Outcomes
FP	Family physicians from pipeline	# counties	29	21	8	7	2	0	
		FP/co.	0	1	2	3	6	>6	70
HPROF	Health professionals produced	# counties	7	11	10	12	13	14	
		HPROF/co.	0	1	2	3	4	5‐12	216
TOTPROF	All professionals^a^ produced	# counties	7	10	11	10	12	17	
		TOTPROF/co.	0	1	2	3	4	5‐12	228
**Exposures**									**Total Exposed**
RMS	Rural medical scholars^b^	# counties	12	10	6	11	6	22	
		RMS/co.	0	1	2	3	4	5‐12	230
RMHS	Rural minority health scholars^c^	# counties	27	7	12	5	2	14	
		RMHS/co.	0	1	2	3	4	5‐20	179
RHS	Rural health scholars^d^	# counties	11	17	13	5	5	16	
		RHS/co.	1‐3	4‐6	7‐9	10‐12	13‐15	16‐24	642

^a^ Health professionals plus PhDs, lawyers, and veterinarians.

^b^ Rural Medical Scholars were rural Alabama students in the 5‐year Rural Community Health (MS or certificate)‐MD program.

^c^ Rural Minority Health Scholars were rural minority Alabama students in a summer program after graduating from high school.

^d^ Rural Health Scholars were rural Alabama students in a summer program after the 11th grade.

Table 2 describes all variables in the study, including their distributions that we discuss below.

**Table 2:** Variable Descriptions and Statistical Distributions From 67 Counties

Continuous Variables				Distribution					
Variable	Type	Definition^a^	Source	Range	Median	Mean	*SD*	Skew.	Kurt.
FP	Outcome	Family physician from pipeline	Program	0‐6	1	1.0	1.3	1.8	4.3
HPROF	“	Health professional produced by pipeline	“	0‐12	3	3.2	2.4	1.1	1.9
TOTPROF	“	All professionals produced by pipeline	“	0‐12	3	3.4	2.6	1.1	1.6
RMS	Exposure	Rural Medical Scholars	“	0‐12	3	3.4	3.0	0.9	0.6
RMHS	“	Rural Minority Health Scholars	“	0‐20	1	2.7	4.1	2.6	7.6
RHS	“	Rural Health Scholars	“	1‐24	8	9.6	6.7	0.7	–0.8
%RURAL	Independent	% of population that is rural	Census^b^	10‐100	71	67.1	26.4	–0.4	–0.9
%POVERTY	“	% of population in poverty	“	8‐39	21	21.5	5.9	0.8	1.2
%BLACK	“	% of population that is black	“	1‐93	24	29.7	23.4	0.9	–0.1
EDUCATION	“	% of adults with ≥ Bachelor's degree (BS)	“	8‐41	14	16.7	7.0	1.7	2.7

Categorical variables				Category 1		Category 2	
Category 1	Category 2			No.	%	No.	%
RURAL	URBAN	Designated rural or urban	ARHA^c^	55	82.09	12	17.91
BLACK BELT	NON‐BB	Designated Black Belt or not	“	18	26.87	49	73.13
EDUC.<17%	EDUC.≥17%	Designated by % of adults with at least BS	Census	46	68.66	21	31.34

^a^ Definitions of intervention and outcome variables are expanded in Table 1.

^b^ US Census Bureau. Reference ^24^.

^c^ ARHA = Alabama Rural Health Association. Reference ^25^.

### Statistical Analyses

We used IBM SPSS Statistics version 26 (IBM Corp., Armonk, NY) for descriptive and hypothesis testing operations, including variable distributions, correlations, and linear regression models, as demonstrated in Tables 2, 3, 4, 5. We followed this analytic sequence:

1. Describe variables, with transformations if necessary.
2. Test for main effects associations between exposure and outcome variables.
3. Screen independent variables for correlates of exposure and/or outcome variables to identify potential control or confounding variables.
4. Test for correlations among variables to identify covariates.
5. Use linear regression to build 3 test models for each outcome by including control variables, first, then testing the addition of each of the 3 exposure variables (*α* ≤ .001).
  Remove from test models control variables that were not significant (*P* ≤ .05) and select the best model for each outcome based on variance explained (*R* ^2^).
6. Use the beta coefficient of the exposure variable in the best model to determine how much the outcome varies with a change of 1 unit in the intervention.

The alpha level was set at .05 to test for associations among variables, at 0.10 to screen for correlates of outcomes to place them in models and, once in, 0.05 to retain them, and at 0.001 to determine statistical significance for exposure variables added to the models in order to account for multiple testing. All *P* values were 2‐sided.

We described continuous variables with range, median, mean, and standard deviation and tested for normality using skewness and kurtosis. We made square root transformations to obtain normality to satisfy assumptions of parametric statistical operations. We produced separate correlations and models with the untransformed and transformed variables. Since there were no differences in the results, we reported the untransformed variable. Similarly, we categorized some variables and compared performance with their continuous counterparts. Categorical variables were shown distributed as percent of counties in each category.

We created statistical models^26^ to explore the associations of exposure variables with outcomes while controlling for selected characteristics of counties (eg, urban/rural, Black Belt/Non‐Black Belt, poverty level, education level, percent Black). We first sought main effect correlations among exposure (eg, RHS, RMHS, and RMS) and outcome variables (eg, TOTPROF, HPROF, and FP) using Pearson's correlation coefficient (*r*).

We next screened for independent variables that could influence main effect correlations to include as control or adjusting variables. We looked among the independent variables for correlates of outcome and/or exposure variables at *P* ≤ .10 using either Pearson's correlation coefficient (*r*) or the *t*‐test. Some characteristics were represented by both continuous and categorical variables. When both of these forms were significantly correlated with outcomes and/or exposures, we chose the continuous variable; otherwise, we chose the significant correlate. We then searched for correlations at *P* ≤ .05 among the chosen independent variables to identify covariates. Where independent variables were covariant, we rejected 1 covariate from the multivariable models. We retained in the model the covariate that added most to the explained variance (*R* ^2^) in outcome.

We made regression models for the 3 outcomes by first including the chosen independent control variables and later adding exposure variables. We did not use earlier exposure program variables (eg, RHS) as independent variables in models testing later exposures (eg, RMS) because of observed overlap among students in these exposure programs (which we validated with assessment of covariance among exposure variables). We tested each pipeline exposure variable (eg, RHS, RMHS, RMS) independently. To produce a model for each outcome, after control variables had been entered we added the exposure variable to test for significant (*P* ≤ .001) correlation and increase in variance explained (*R* ^2^) of the outcome. This process produced 3 test models for each outcome. We produced the best model for each outcome by removing from the 3 exposure test models any control variables whose contributions were not statistically significant (*P* ≤ .05), then comparing the resulting *R* ^2^s.

## Results

### Program Participation and Outcomes

Table 1 describes the distribution among counties of outcomes and participation for the 3 exposure programs. All 67 counties had 642 RHSs, 40 counties had 179 RMHS, and 55 counties had 230 RMSs. Sixty counties produced at least 1 unspecified professional (TOTPROF) for a total of 228 and at least 1 health professional (HPROF) for a total of 216. Thirty‐eight counties received 70 family physicians (FP).

### Data Distributions and County Descriptions

Table 2 provides statistical descriptions of variables in the study. We sought continuous variables to conserve statistical power. Data from the Census^24^ supplemented program data: %RURAL from the Census includes all persons who live outside of an Urbanized Area of ≥50,000 people or Urban Clusters of 2,500 to 50,000^27^; %BLACK refers to Black or African Americans whose origins include any of the Black racial groups of Africa^28^; %POVERTY includes those whose family income meets the Census poverty threshold that is adjusted geographically using the Consumer Price Index^29^; and EDUCATION derives from the Census Education Attainment tables showing population of 25 years and older who attained at least a bachelor's degree.^30^ Two continuous variables (eg, FP and RMHS) were not normally distributed but demonstrated normality after square root transformation. However, there were no differences in performance in models with their untransformed or transformed states, so we provided their untransformed statistics. Counties’ mean percentage of population rural was 67%, Black 30%, in poverty 22%, and adults with at least a bachelor's degree 17%.

Three variables (eg, %RURAL, %BLACK, and EDUCATION) were made categorical to facilitate analysis. These categorical variables were labeled RURAL/URBAN, BLACK BELT/NON‐BB, and EDUC.<17%/EDUC.>17%. The Alabama Rural Health Association used Metropolitan Statistical Areas to designate 12 counties urban and 55 counties rural^25^; among the rural counties we grouped 18 as Black Belt.^18^ Forty‐six counties had less than 17% of adults with at least a bachelor's degree.

### Correlations Among Exposures and Outcomes

Table 3 shows the main effects association of each pipeline exposure program and studied outcomes. RMS participation by a county was correlated with receiving a family physician from the pipeline (*r* = 0.55, *P* < .001), producing health professionals (*r* = 0.48, *P* < .001), and producing all professionals (*r* = 0.39, *P* = .001). RHS participation also was significantly correlated (*P* ≤ .002) with each of the 3 outcomes, *r* = 0.38, 0.51, and 0.54, respectively. RMHS participation correlated with production of all professionals (*r* = 0.25, *P* = .04).

**Table 3:** Bivariate Pearson Correlations Among Exposure and Outcome Variables

	Outcomes					
	FP		HPROF		TOTPROF	
Exposure Pipeline Program	*r*	(*P)*	*r*	(*P*)	*r*	(*P*)
RMS	0.55	(<.001)	0.48	(<.001)	0.39	(.001)
RMHS	0.02	(.86)	0.20	(.10)	0.25	(.04)
RHS	0.38	(.002)	0.51	(<.001)	0.54	(<.001)

We also found correlations among exposure variables—between RHS and RMHS (*r* = 0.54, *P* < .001) and between RHS and RMS (r = 0.32, *P* = .008). We restricted later multivariable models to include no more than 1 of these covariant exposure variables.

### Screening of Independent Variables

Table 4 demonstrates those independent variables that screened positive as potential confounders or as control variables to adjust the main effects associations shown above. %POVERTY, %BLACK, BLACKBELT +/–, and RURAL/URBAN had no significant relationship with the outcomes, but each was related (*P* ≤ .10) with 2 of 3 program exposures. EDUCATION and EDUCATION<17%/≥17% had no significant relationship with outcomes and 1 with an exposure (eg, RHS). %RURAL did not show a relationship with any outcome or exposure.

**Table 4:** Screening of Independent Variables for Relationships With Outcome and/or Exposure Variables (Using *r* or *t*)^a^ at *P* ≤ .10

Independent Variables	Outcome Variables						Exposure Variables					
	FP		HPROF		TOTPROF		RMS		RMHS		RHS	
Continuous	*r*	(*P*)	*r*	(*P*)	*r*	(*P*)	*r*	(*P*)	*r*	(*P*)	*r*	(*P*)
%RURAL	0.00	(.98)	–0.14	(.26)	–0.16	(.20)	–0.15	(.23)	0.16	(.21)	0.16	(.20)
%POVERTY	–0.10	(.42)	0.06	(.62)	0.12	(.35)	–0.26	(.04)	0.37	(.002)	0.19	(.12)
%BLACK	–0.12	(.32)	0.03	(.80)	0.09	(.49)	–0.41	(.001)	0.34	(.004)	–0.03	(.80)
EDUCATION	–0.05	(.67)	0.06	(.65)	0.07	(.57)	0.07	(.58)	–0.12	(.35)	–0.22	(.08)

Categorical	*Mean*	(*P*)	*Mean*	(*P*)	*Mean*	(*P*)	*Mean*	(*P*)	*Mean*	(*P*)	*Mean*	(*P*)
RURAL	1.2	(.18)	3.1	(.49)	3.3	(.54)	3.3	(.35)	3.1	(.09)	10.4	(.04)
URBAN	0.6		3.7		3.8		4.2		0.8		6.1	
BLACKBELT	0.8	(.32)	3.1	(.74)	3.5	(.86)	1.7	(.003)	4.8	(.01)	9.9	(.79)
NON‐BLACKBELT	1.1		3.3		3.4		4.1		1.9		9.4	
EDUCATION<17%	1.1	(.56)	3.2	(.94)	3.4	(.96)	3.4	(.73)	3.1	(.20)	10.8	(.02)
EDUCATION≥17%	0.9		3.2		3.4		3.6		1.7		6.9	

^a^ “*r*” for Pearson's correlation coefficient; “*t*” for *T*‐test (used with the categorical independent variables).

There was covariance among independent variables. There were strong correlations between EDUCATION and both %RURAL (*r* = –0.78, *P* < .01) and %POVERTY (*r* = –0.44, *P* < .01). There was also correlation between %POVERTY and both %BLACK (*r* = 0.60, *P* < .01) and %RURAL (*r* = 0.36, *P* < .01).

### Linear Regression Models

Table 5 shows multivariable linear regression models built to assess the role of pipeline exposure programs in influencing outcomes while adjusting for other factors. The table shows variance explained in outcomes (*R* ^2^) by control variables in the model first (control model), then by the added pipeline exposure variables taken individually (test models). The exposure variable with greatest contribution to *R* ^2^ is shown in the best model.

**Table 5:** Models Testing Pipeline Exposures and Providing Best Explanation for Variance of Outcomes (*R* ^2^)

	Control Variables						Test Exposure Variables						*R* ^2^
	%POVERTY		%BLACK		%RURAL		RHS		RMHS		RMS		
Outcome Modeled	*b*	(*P*)	*B*	(*P*)	*b*	(*P*)	*b*	(*P*)	*b*	(*P*)	*b*	(*P*)	
TOTPROF													
Control model	0.10	.18	–0.004	.80	–0.02	.08	–^a^	–	–	–	–	–	0.06
Test RHS model	0.02	.73	0.01	.52	–0.03	.02	0.22	<.001	–	–	–	–	0.36
Test RMHS model	0.07	.32	–0.01	.55	–0.02	.06	–	–	0.16	.053	–	–	0.12
Test RMS model	0.08	.21	0.02	.24	–0.02	.19	–	–	–	–	0.43	<.001	0.26
Best model	–	–	–	–	–0.02	.02	0.23	<.001	–	–	–	–	0.35
HPROF													
Control model	0.07	.32	–0.01	.69	–0.02	.15	–	–	–	–	–	–	0.04
Test RHS model	0.002	.98	0.01	.69	–0.02	.05	0.20	<.001	–	–	–	–	0.31
Test RMHS model	0.05	.48	–0.01	.48	–0.02	.11	–	–	0.14	.09	–	–	0.08
Test RMS model	0.05	.38	0.02	.19	–0.01	.36	–	–	–	–	0.47	<.001	0.30
Best model	–	–	–	–	–0.02	.04	0.20	<.001	–	–	–	–	0.31
FP													
Control model	–0.01	.77	–0.01	.56	0.001	.85	–	–	–	–	–	–	0.02
Test RHS model	–0.04	.28	0.00	.98	0.00	.99	0.08	.001	–	–	–	–	0.17
Test RMHS model	–0.02	.69	–0.01	.50	0.001	.88	–	–	0.03	.54	–	–	0.02
Test RMS model	–0.02	.50	0.01	.20	0.01	.29	–	–	–	–	0.28	<.001	0.33
Best model	–	–	–	–	–	–	–	–	–	–	0.24	<.001	0.30

^a^ “—” indicates that the variable was not included in the model.

We dropped education from the analysis as a covariant. After controlling for poverty, race, and rurality, we found that counties with more RHSs and RMSs produced more total professionals and health professionals and gained more family physicians from the pipeline. Counties with more RMHSs did not produce more professionals at alpha equal .001. The best models for county production of total professionals (TOTPROF) and health professionals (HPROF) included number of RHSs, with respective beta coefficients of 0.23 (*P* < .001) and 0.20 (*P* < .001), and percent rural with respective *R* ^2^s of 0.35 and 0.31. In both models, percent rural was negatively associated with the outcome with beta coefficients in each case of –0.02 (*P* ≤ .05). Neither percent in poverty nor percent Black reached significance. In the total professionals model, an increase by 1 Rural Health Scholar was associated with an increase in 0.23 professionals or 1 professional for 4 RHSs. Similarly, the health professional model showed 1 health professional for 5 RHSs.

The best model for county acquisition of family physicians from the pipeline included only the county's number of RMSs (*b* = 0.24, *P* <.001) with *R* ^2^ of 0.30. For 4 RMSs, a county's family physicians increased by 1.

In summary, counties that populated the pipeline with more rural high school and graduate/professional students produced more professionals and gained more family physicians.

## Discussion

The more a county participated in these programs, the greater its return in terms of physicians gained and health professionals produced. The *R* ^2^s of 0.35, 0.31, and 0.30 for professionals, health professionals, and family physician models, respectively, indicate that RHLP programs have higher than moderate ability to explain variance in these outcomes.^31^ The effects were not trivial in that a county gained 1 family physician for 4 RMS and produced 1 other health professional for 5 RHS.

In this study, we measured community participation by the number of local students in the pipeline programs; however, this was an end result of recruiting efforts that reached out to schools, social groups, and local government officials. These various recruitment targets responded in a variety of ways. Our unstructured observations give us the impression that participation was greater among communities that endorsed these programs with enthusiasm and invested time, energy, and resources to both encourage their students and assist the programs by providing community activities, fieldtrips, and role models. Anecdotally, we heard from various community officials and parents about encouraging their state representatives to strengthen the funding stream for these programs.

A singular strength of the study was using county as the unit of observation. Counties are policy units where community needs often meet social action. Local officials can relate county data to their influence in the process of involving students in pipelines to produce professionals and gain family physicians. The payoff is in culturally consonant health care from locally grown health professionals. This suggestion is in line with another study from Alabama that found counties with more students in medical school had more primary care physicians and longer life expectancy.^32^ As counties involve more students in these pipelines, we could expect them also to seek ways to better attract these pipeline students into local practice. These efforts might be internal to the community, such as local scholarships, shadowing opportunities, social recognition, and civic involvement. Local leaders might advocate externally for a reform that brings professional education of these students into their communities (eg, community‐based, distributed education) and for policies to support their future practice there, eg, service‐based financial aid, tax breaks, professional networking, and local hospital support. It would be but a small additional step for such an activated community to expect and seek further reform through engagement in the planning, conduct, and monitoring of resulting educational, placement, and practice programs. A process of rural health care reform similar to this is being pursued in Northern Ontario.^33^

In addition to increasing rural workforce, the RHLP could promote other reforms in medical education. Though this article is not meant to review the sphere of medical education reforms that are discussed elsewhere,^3^, ^4^ we suggest that involved local government officials and their communities can provide a potent impetus to reform by focusing state educational and health care policies on the health and well‐being of the population. Using the improvement of local populations’ health as the criterion for governmental support could stimulate many reforming efforts. The RHLP is one example of a local reform, which leads to consideration of additional reforms that advance toward community health. For example, the diversity of professional aspirations among each class of pipeline students provides preformed cohorts of students readymade for interdisciplinary and interprofessional training.^11^ Also, longitudinal observations of pipeline participants’ performance and character create opportunity to assess their ability to succeed in advanced health professional education aside from use of standardized tests.^16^, ^34^, ^35^, ^36^, ^37^ This alternative assessment could offset an unintentional exclusion of capable rural, minority, and nontraditional students that is linked to such tests.^33^ These local reforms could be evaluated based on their effects on the health of targeted underserved rural populations.

### Limitations

This study is limited for causal analysis by the observational design and limited number of counties (N = 67). The small number affected our ability to detect associations with the RMHS program, which because of briefer existence included only 40 counties, and limited the number of control variables that could be considered. The data included family physicians’ initial practice location and not longevity, thus restricting the study's applicability to recruitment and not retention.

Demographic diversity among the studied counties is a strength; however, generalizability may be an issue. The data generate from 1 southern state. We have shown previously that the program produces rural physicians among Alabama's White‐dominant counties similar to programs in states with White majorities.^14^ However, we have no comparison for the 18 Black‐dominant counties.

## Conclusion

We conclude that these data support the hypothesis that exposing more local students in rural pipelines produces desired results for participating counties. In Alabama, for every 4 RMS students in the pipeline, a county gained 1 family physician. We encourage community leaders to increase the number of their students in rural pipelines, advocate for pipeline program support, and engage in policy processes aimed at improving population health. Also, we recommend that evaluation studies accompany pipelines developed in different contexts to explore additional factors that influence pipeline effectiveness. We suppose that collaboration between local communities and institutions to produce and populate rural pipelines is only one central component of a larger mosaic of actions among communities, institutions, governmental agencies, insurers, and philanthropies that will increase and sustain health professionals in underserved rural communities. Governmental priority to population health driven by pressure from local officials and constituencies will be required to piece together this mosaic, with Australia's plan^17^ serving as a national example and the Northern Ontario School of Medicine showing a provincial response.^33^ We call on local public officials throughout the underserved rural regions of the United States to advocate for comprehensive systems of education and health care tailored to the health disparities among their populations. An early policy target might be a program of demonstration research projects to build effective systems of health care among significantly underserved rural groups with concentrated health disparities such as African Americans in the South, Hispanics in the Southwest, Native Americans in the West, and White Americans in Appalachia.^38^, ^39^, ^40^ These research projects would document and monitor the developmental processes, account for the human and material resource requirements, and evaluate the attainment of anticipated milestones and outcomes. With a departure from traditional medical education and health care in order to engage the communities and achieve their good health, we should anticipate the research to take a holistic interdisciplinary perspective including expertise with culture, sociology, psychology, education, and health services research.
